# Novel and Investigational Treatments for Onychomycosis

**DOI:** 10.3390/jof8101079

**Published:** 2022-10-14

**Authors:** Stamatios Gregoriou, Maria Kyriazopoulou, Aikaterini Tsiogka, Dimitrios Rigopoulos

**Affiliations:** Department of Dermatology-Venereology, Faculty of Medicine, Andreas Sygros Hospital, National and Kapodistrian University of Athens, 16121 Athens, Greece

**Keywords:** onychomycosis, topical, antifungals, nail, treatment, Efinaconazole, Tavaborole, Luliconazole, Terbinafine, Amphotericin B, ME-1111, NP213

## Abstract

Onychomycosis is a common nail disease caused by fungi. The primary pathogens are dermatophytes; however, yeasts, non-dermatophyte moulds, and mixed fungal populations may also contribute to the development of a recalcitrant condition, usually accompanied by difficulties in everyday life and severe emotional stress. Treatment failure and relapse of the infection are the most frequent problems, though new issues have become the new challenges in the therapeutic approach to onychomycosis. Resistance to antifungals, an increasing number of comorbidities, and polydrug use among the ageing population are imperatives that impose a shift to safer drugs. Topical antifungals are considered less toxic and minimally interact with other drugs. The development of new topical drugs for onychomycosis is driven by the unmet need for effective agents with prolonged post-treatment disease-free time and a lack of systemic impact on the patients’ health. Efinaconazole, Tavaborole, and Luliconazole have been added to physicians’ weaponry during the last decade, though launched on the market of a limited number of countries. The pipeline is either developing new products (e.g., ME-1111 and NP213) with an appealing combination of pharmacokinetic, efficacy, and safety properties or reformulating old, well-known drugs (Terbinafine and Amphotericin B) by using new excipients as penetration enhancers.

## 1. Introduction

Onychomycosis is a term that encompasses all the nail pathologies caused by fungi and accounts for approximately 50% of all nail diseases [1]. The reported mean prevalence in Europe and North America is 4.3–8.9% [2]. The epidemiologic data for onychomycosis exhibit remarkable geographical variance, depicting differences regarding the ecology of fungi and the characteristics of the affected population (i.e., genetics, culture, climate, and lifestyle). In Western countries, the primary culprit pathogens are dermatophytes (up to 80–90% of cases), whilst yeasts and non-dermatophyte moulds (NDMs) account for 5–17% and 2–3% of cases. The prevalence of dermatophyte infection is lower in South Europe and Asia/the Middle East, accounting for 40–68% and 40–48%, respectively. Yeasts are isolated in 21–55% of cases in South Europe and 43–46% in the Middle East. NDMs attribute to 8–11% of infections in Asian countries [3]. According to a recent Iranian report [4], samples collected by two laboratories affiliated with Tehran University exhibited 33.7%, 21.8%, and 44.4% rates for yeast, dermatophyte, and NDM onychomycoses, respectively. This is a remarkably different epidemiologic pattern compared to that demonstrated in the Western world.

For dermatophyte onychomycosis, *tinea pedis* is usually a precondition, and a familial pattern was described by Zaias et al. in 1996, postulating a genetic basis in the susceptibility of developing the infection [5]. NDM onychomycoses are opportunistic infections and are typically characterised by the absence of concomitant interdigital space infection. Exposure to NDMs may lead to onychomycosis in the presence of numerous predisposing factors [6]: high temperatures and humidity due to climate conditions, occlusive footwear and hyperhidrosis; advanced age; nail deformity due to acute or chronic trauma; chronic cutaneous diseases, such as psoriasis; comorbidities with impaired vascular and immune function; environmental exposure due to occupational or leisure activities; toenail damage presenting as asymmetric gait nail unit signs (AGNUS) [7,8,9]. The latter provides a plausible explanation for the formation of a nail unit space, which is colonised by opportunistic fungi and serves as a niche for the constant presence of the NDM in the toenail environment. Therefore, it can be extrapolated that the infection is not the primary one in a sequence of events which usually starts with the formation of a cleft between the nail plate and bed, generated by chronic or acute trauma and habitual or occupational injury. Mixed infections by dermatophytes and non-dermatophytes are increasing in prevalence, either on the basis of impaired anatomy or not. They establish a refractory microenvironment, which necessitates a thorough assessment in terms of clinical and laboratory evaluations [10].

Onychomycosis has a severe impact on patients’ quality of life and poses a challenge to doctors’ practice. Onychomycotic nail deformity and dystrophy often present as both aesthetic and functional disorders that may result in difficulty in proper shoe fitting and subsequent inconvenience or even pain during daily activities. Treatment failure has been reported in 20–50% and recurrence rates in 10–53% of the cases [11,12,13]. Socks and shoes comprise the main reservoirs of fungi and sources of reinfection. The nail growth rate is low, and treatment, either systemic or topical, is required over long periods of time. Compliance with long-term treatments is often poor. Adherence to systemic therapy for months is a practice that increases safety and drug-to-drug interaction issues, especially in the case of elderly patients or patients with multiple comorbid clinical entities. Topical treatments of onychomycosis, used either as a monotherapy or adjuvant therapy, are generally characterised by low efficacy. The drug concentration in the tissue is highly dependent on the permeability/penetration qualities of the topically applied agents. Improving transungual distribution is considered as one of the key targets for the development of effective new agents. As mentioned above, the resistance to antifungal medicines is another emerging issue, adding further difficulty to the management of onychomycosis. Consequently, an unmet need for more efficacious active molecules, vehicle formulations to enhance penetration, and improved treatment algorithms, particularly regarding long-term prophylaxis, exists.

## 2. Materials and Methods

### 2.1. Novel Topical Antifungal Agents

**Scheme 1:**
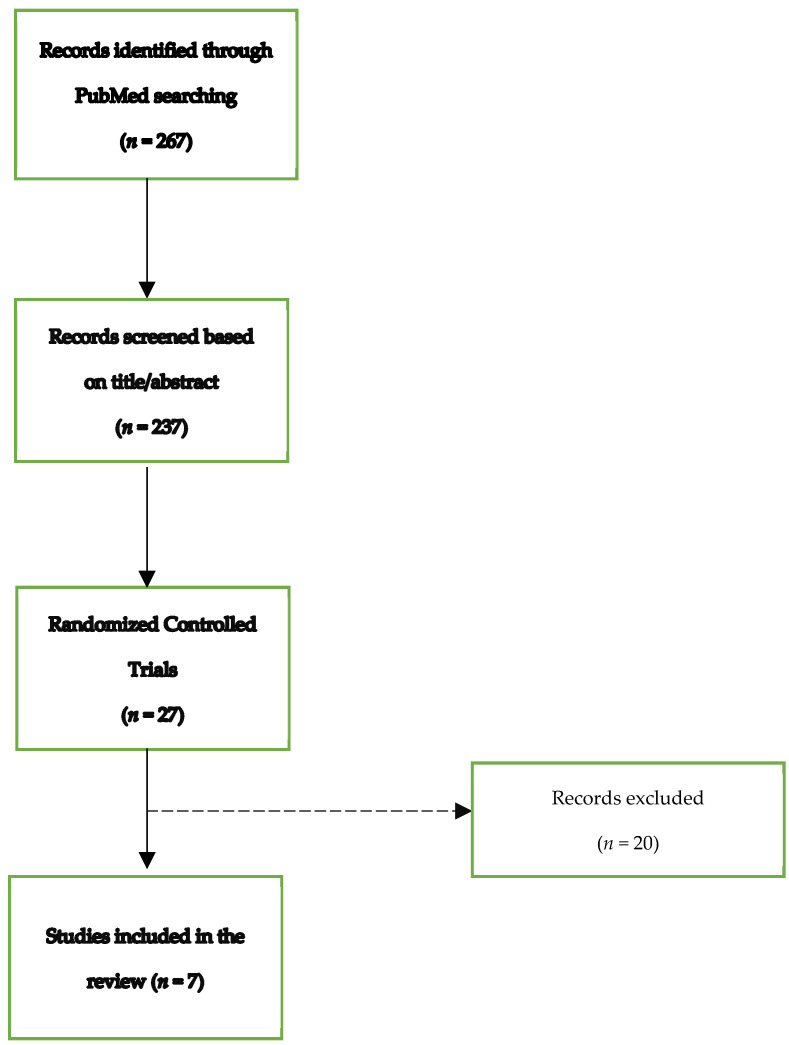
Flow diagram of the PubMed search.

The MEDLINE database was searched systematically via PubMed for randomised controlled trials (RCTs) using the terms “onychomycosis”, “topical”, “antifungal”, “Efinaconazole”, “Tavaborole”, and “Luliconazole”. This review was performed according to Preferred Reporting Items for Systematic reviews and Meta-Analyses (PRISMA). A summary of the search strategy is set out in Table 1. Non-human studies were excluded *ab initio*. The search yielded 267 articles initially and 237 articles after a title/abstract search was performed. Inclusion criterion: randomised controlled trials (RCTs). Twenty-seven studies were selected for full-text review by two independent authors (M.K. and A.T.). Exclusion criteria: irrelevant records, post hoc analyses of data derived from pivotal studies, studies assessing the combination of topical antifungal and laser treatment, and studies referring to the combination of onychomycosis and tinea pedis. Twenty studies were excluded, and seven studies were finally included in this review (Scheme 1 and Table 2). 

### 2.2. Antifungal Drugs under Investigation

The ClinicalTrials.gov database of the U.S. National Library of Medicine and the EU Clinical Trials Register were screened for topical antifungal drugs under investigation using the searching terms “onychomycosis” and “topical”. Study phase and recruitment status (recruiting, active, completed, and unknown) filters were applied. Inclusion criterion for further review: RCTs presenting with their last update within the last decade. Exclusion criterion: agents with insufficient information about their chemical structure or mechanism of action. All the RCTs reporting the efficacy and safety of novel and investigational topical antifungal agents that met the search criteria are listed in Table 2 and Table 3, respectively.

## 3. New Topical Agents for Onychomycosis

The topical agents that have been most recently approved include Efinaconazole, Tavaborole, and Luliconazole. Data published on their safety and efficacy come from a limited number of countries where the drugs have become available.

### 3.1. Efinaconazole

**Figure 1:**
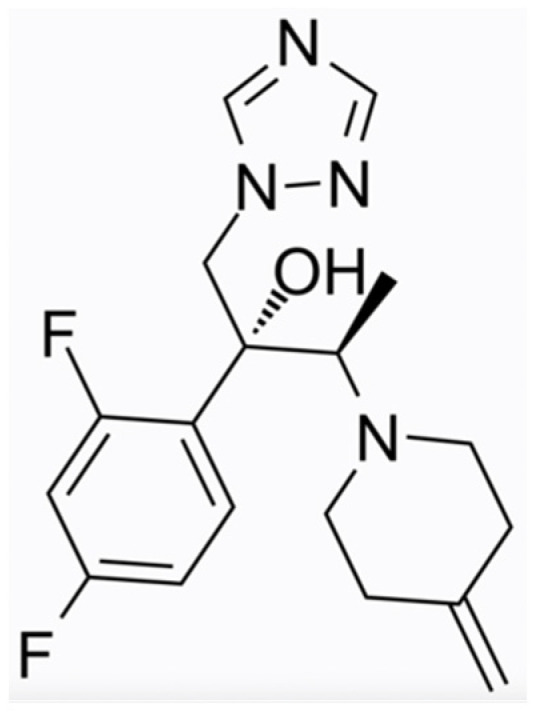
Efinaconazole chemical structure.

Efinaconazole (molecular formula C18H22F2N4O, chemical structure as seen in Figure 1), a next-generation triazole, has been available in the USA and Japan since 2014. It is FDA-approved for the treatment of onychomycosis due to *T. rubrum* and *T. mentagrophytes* for patients aged 18 years and older. In 2020, this indication was extended to children 6 years of age and older. It is administered as a 10% solution and blocks ergosterol synthesis by inhibiting the enzyme lanosterol 14-dimethylase. Nail penetration of Efinaconazole is enhanced by an efficient combination of physicochemical properties, such as low surface tension, low affinity to keratin, and poor aqueous solubility [25]. Efinaconazole displays a low interaction with the nail keratin, as measured in bovine hoof membranes, a widely used model for the human nail [26]. Accordingly, it has been demonstrated that the site of action for this drug is the nailbed and fails to achieve a high concentration within the nail plate. In two phase III randomised, double-blinded studies, Efinaconazole 10% solution exhibited mycological cure rates (negative potassium hydroxide, KOH, direct examination and negative culture) of 55.2% and 53.4% in patients with distal–lateral subungual onychomycosis after treatment for 48 weeks (superior to vehicle, *p* < 0.001). Complete cure rates (mycologic cure and absence of clinical involvement) occurred in 17.8% and 15.2% vs. 3.3% and 5.5% in the vehicle-receiving group of subjects [15]. A retrospective survey of the treatment results documented a 25% complete cure rate in patients with severe involvement (Scoring Clinical Index for Onychomycosis, SCIO = 21–30) [27]. Efinaconazole is fungistatic, not fungicidal; therefore, it may induce resistance in dermatophytes such as other azole derivatives. Actually, it has been observed that *T. rubrum* strains with resistance to Efinaconazole may show cross-resistance to Itraconazole but not Amorolfine or Ciclopirox [26].

### 3.2. Tavaborole

**Figure 2:**
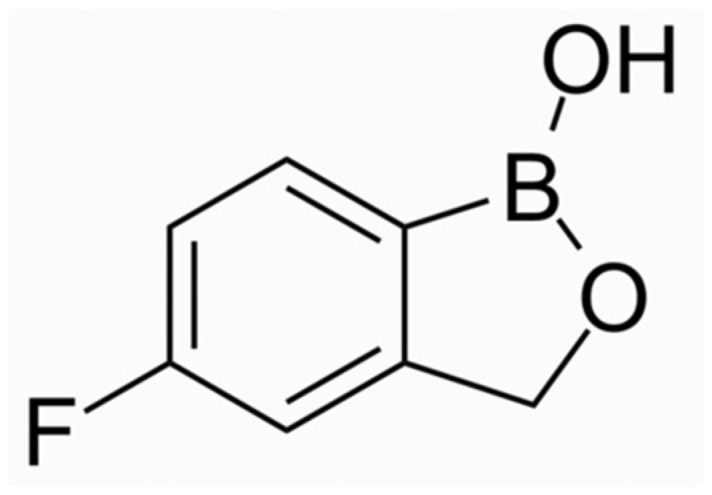
Tavaborole chemical structure.

Tavaborole (molecular formula C7H6BFO2, chemical structure as seen in Figure 2) has been US FDA-approved since 2014. This oxaborole drug inhibits the enzyme leucyl-tRNA synthetase and interferes with the process of protein synthesis, thus exerting its action to a broad spectrum of microorganisms, i.e., fungi (dermatophytes, yeasts, and non-dermatophyte moulds) and some bacteria. The efficacy and safety of tavaborole were evaluated in two identical phase III multicentre, randomised, double-blinded, vehicle-controlled trials [18]. Adults with distal subungual onychomycosis that affected 20–60% of the great toenail were enrolled for a 48-week intervention with once-daily applications. An assessment of the results was performed at week 52. Tavaborole solution 5% was significantly superior to the vehicle in all endpoint efficacy parameters. The complete cure rates for the target great toenail were 6.5% and 9.1% for Tavaborole vs. 0.5% and 1.5% for the vehicle (*p* = 0.001 and *p* < 0.001), respectively. The mycological cure rates (negative culture and KOH direct microscopy) were 31.1% and 35.9% for tavaborole-treated patients vs. 7.2% and 12.2% for the vehicle group (*p* < 0.001 for both), respectively. For both studies, treatment-related adverse events were higher in the Tavaborole arms, but the discontinuations attributed to them were almost equal. The low molecular weight (152 Da) of Tavaborole offers a clear penetration advantage and results in enhanced distribution through the nail plate compared to Ciclopirox 8%, as was demonstrated by Hui et al. [28]. It has been assumed that Tavaborole is not likely to induce resistance owing to its mechanism of action [29]. However, the in vitro resistance of *T. rubrum* strains and isolates to Tavaborole has been developed by propagating them on Sabouraud dextrose agar media containing low, non-inhibitory concentrations of the drug (0.5 × MIC) [30]. As mentioned above, the favourable pharmacokinetic profile of Tavaborole contributes to high concentrations in the ungual apparatus in vitro, but it is unknown whether host or disease-related factors could reverse this merit, leading to the development of resistant strains in vivo. Moreover, a low efficacy of Tavaborole against yeasts and moulds compared to Terbinafine and azoles (except Fluconazole) has been reported [31].

### 3.3. Luliconazole

**Figure 3:**
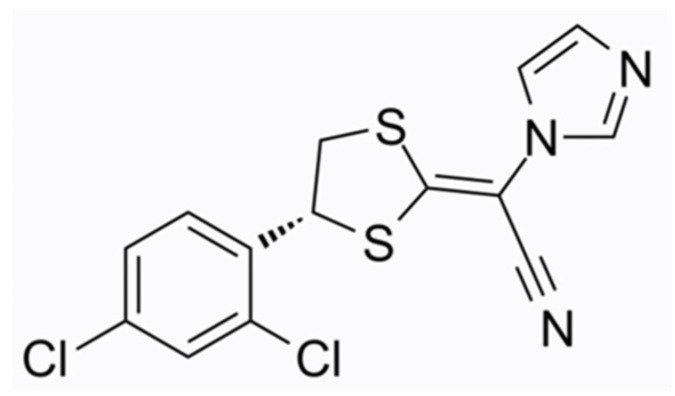
Luliconazole chemical structure.

The most recently approved antifungal topical agent for onychomycosis is Luliconazole 10% solution (molecular formula C14H9Cl2N3S2, chemical structure as seen in Figure 3), a member of the imidazole subfamily, which became available in Japan in 2016. Even though the FDA has approved Luliconazole cream 1% for the treatment of tinea pedis, tinea cruris, and tinea corporis, approval of the solution for onychomycosis is pending in the USA. The molecular target of Luliconazole is lanosterol 14a-demethylase, an enzyme involved in the biosynthesis of fungal cell membranes, which is inhibited by the drug [20,32]. Luliconazole is administered on the nail as a 5% *w*/*w* solution. The drug concentration increases in a dose-dependent manner, as it is positively associated with the duration of application. Luliconazole penetrates the nail plate and rapidly achieves fungicidal levels in the nail unit independent of the nail plate thickness [33]. Luliconazole was reported to achieve a 14.9% complete cure rate (0% clinical involvement and negative KOH test under direct microscopy), which was significantly higher compared to the vehicle (5.1%). The efficacy was evaluated in a multi-centre, double-blinded, randomised phase III study after 48 weeks of daily application in adults with distal–lateral subungual onychomycosis with 20–50% clinical involvement of the great toenails [20]. Shimoyama et al. documented a complete cure rate of 15.8% in patients with SCIO 21-30 affected by various types of onychomycoses (superficial white onychomycosis, distal–lateral subungual onychomycosis, proximal subungual onychomycosis, and dermatophytoma) after long-term treatment (mean duration 12.4 months) [27]. The safety and efficacy of luliconazole 10%, not 5%, solution is being evaluated in a currently active, open-label, phase I study in patients with moderate to severe distal subungual onychomycosis (NCT05110638). The different approach is that the application is performed by the study personnel once daily for 29 consecutive days to all toenails and periungual areas, regardless of whether they are affected or not.

## 4. Topical Antifungal Nail Therapies under Development and Investigation

The onychomycosis pipeline holds investigational topical drugs endowed with favourable transungual penetration profiles, high efficacy, and biostability, resulting in a prolonged disease-free status within the nail apparatus.

### 4.1. ME-1111

**Figure 4:**
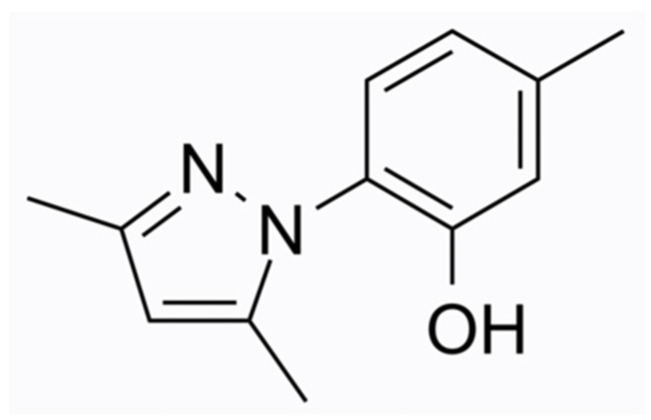
ME-1111 chemical structure.

ME1111 (molecular formula C12H14N20, chemical structure as seen in Figure 4) is a new antifungal drug under clinical development as an onychomycosis treatment (NCT01841996 and NCT02022215). It has a relatively low molecular weight and decreases ATP production in dermatophyte mitochondria by the inhibition of succinate dehydrogenase (complex II) [34]. The pharmacokinetic parameters of ME-1111 were determined by an in vitro study compared to Efinaconazole, Tavaborole, Ciclopirox, and Amorolfine [35]. ME-1111 (10% sol. *w*/*v*) and Tavaborole (5% *w*/*w*) attained high concentrations in the deep nail layers due to their low molecular weights (202.25 Da and 152 Da, respectively) and low keratin-binding affinity, which both render permeability easier. Ciclopirox (in an 8% *w*/*v* formulation), albeit light (molecular weight 207.27), displayed low concentrations in the deep nail plate, a result that was attributed to its high keratin-binding affinity. The in vitro anti-dermatophytic efficacy coefficient (i.e., total or drug-free concentration in the nail plate/MIC90) against *T. rubrum* was estimated at pH 5.0 and pH 7.0. For both environments, this parameter exceeded 11,000 in the total drug and 6000 in the drug-free measurements, providing evidence of possible strong anti-dermatophyte potency in the nail. The differences in the drug-free efficacy coefficient values between ME-1111 and the other topical antifungals were statistically significant. These outcomes displayed consistency with the data shown in an onychopharmacokinetic study by Hui et al. [36]. According to this, a 10% ME-1111 topical formulation was significantly superior to 8% Ciclopirox nail lacquer regarding the penetration and attainment of high concentrations in the deeper nail layers. The coexistence of favourable pharmacokinetic and efficacy properties composed a promising and attractive drug profile. Further investigation by high-quality in vivo studies is required. 

### 4.2. NP213

**Figure 5:**
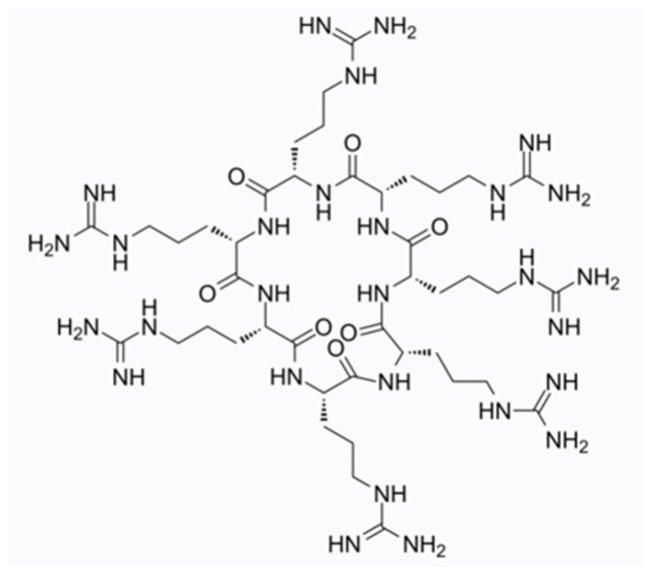
NP213 chemical structure.

NP213 (NVXT or Novexatin, molecular formula C42H84N28O7, chemical structure as seen in Figure 5) is a new topical synthetic peptide under investigation for the treatment of onychomycosis. It exerts its activity by a novel unique mechanism, as it has been designed and developed based on the host defence peptide (HDPs, also called antimicrobial peptides, AMPs) structure and pattern of action. NP213 consists of seven arginine amino acids arranged in a highly hydrophilic cationic cyclic peptide [37]. The positively charged peptide rapidly penetrates the negatively charged nail and circumvents the proteolytic activity of the dermatophyte peptidases and proteases due to its all-arginine composition and cyclic structure. According to Mercer et al. [37], NP213 expressed both antihyphal and sporicidal activity against *T. rubrum* after 3–4 h of incubation, whereas Terbinafine failed to kill any dermatophyte form (germlings or spores) in 24 h, although tested in a high (2 × MIC) concentration. Studying the NP213 mode of action by scanning electron microscopy (SEM) revealed a flatter appearance of hyphae after 48-h exposure, which indicated a loss of the cytoplasmic volume and internal cellular turgor. On the contrary, SEM images following incubation with Terbinafine and Ciclopirox appeared similar to images of untreated cells. Further investigation with transmission electron microscopy (TEM) revealed a loss of organelles after exposure for 6, 18, and 48 h to 10×MIC NP213 concentrations compared to the control cells, within which the well-visible organelles appeared intact. It is assumed that NP213 probably causes the lysis of cytoplasmic membranes without affecting the cell wall integrity. Assessment of the activity and biostability of NP213 in an ex vivo nail model was performed for up to 11 months following a 28-day application. NP213-cleared nails were re-exposed to *T. rubrum* at timepoints of 3, 5, 8, and 11 months after the end of the treatment period and compared to healthy control nails. No evidence of reinfection was demonstrated in the NP213 group, and this was statistically significant compared to the controls at every timepoint. Two studies evaluating the in vivo safety and efficacy of the NP213 regimen of 28 daily applications have been conducted [21]. In the first one (a phase I/IIA randomised, placebo-controlled, two sequential parts trial), adults with 25–75% nail involvement and undetermined fungal pathogens were enrolled. The outcomes reported negative cultures at 43.3% after 180 days. Similarly, the results in the second trial (a phase IIA randomised, double-blinded, placebo-controlled pilot study) stated that 56.5% of the patients with 10–50% toenail involvement due to dermatophyte infection remained culture-negative for 360 days post-treatment. It is evident that NP213 has the potential to become a useful therapeutic tool in the future, a drug with high efficacy and biostability that establishes a disease-free microenvironment within the nail apparatus. 

### 4.3. Topical Terbinafine

**Figure 6:**
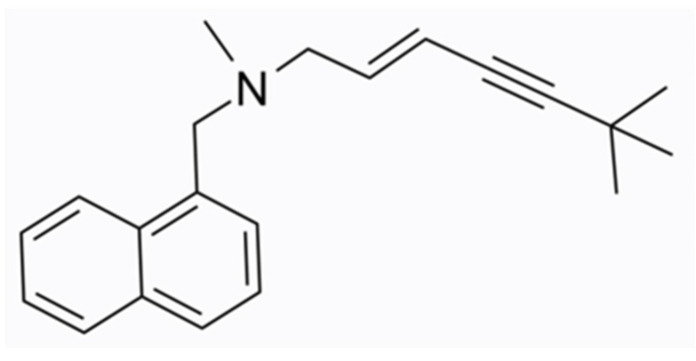
Terbinafine chemical structure.

The evaluation of topical terbinafine (molecular formula C21H25N, chemical structure as seen in Figure 6) in various formulations for the treatment of onychomycosis has been based on the long-term use of oral terbinafine as a fungicidal with a reliable efficacy and safety profile in clinical practice. The published data report mycological and complete cure rates of 70% and 38%, respectively [38]. MOB015B is a Terbinafine 10% solution for the topical treatment of onychomycosis. Its efficacy and pharmacokinetics were assessed in a phase IIA study (NCT01814020) showing remarkably higher concentrations in the nail plate (median value 1610 μg/g) and nailbed (median value 45 μg/g) than oral Terbinafine (up to 1000-fold and 40-fold, respectively) [39]. In terms of safety, the detected plasma concentrations after 4 weeks of exposure were very low. The mycological cure was measured at week 60 after a 48-week regimen with daily applications and was reported negative in 54.2% of the patients who completed the trial. In a phase III study [22], patients 12–75 years old with distal subungual onychomycosis (dermatophyte infection previously confirmed by a positive culture) involving 20–60% of at least one great toenail were enrolled. The mycological cure rates at week 52 were 69.9% and 27.7% for MOB015B subjects and the vehicle group, respectively (*p* < 0.001). Complete cure rates were much lower: 4.5% for the drug and 0% for the vehicle. There was no difference when oral and topical agents were compared in terms of the mycological cure rates, but the values regarding a complete cure did not demonstrate any proximity. A possible explanation could be that a complete cure encompasses the clinical cure parameters, which are susceptible to subjective judgment. The MOB015B formula contains urea, lactic acid, and propylene glycol as enhancers of penetration. These agents contribute to the appearance of an opaque discoloration, and therefore, the impression of a persistent infection is falsely given. The negative cultures ranged from 93.5% at week 12 to 95.9% at week 52. These results suggest not only an early onset of fungal eradication, probably due to high Terbinafine levels achieved in the nail (a thousand times greater than the MIC for the usual dermatophyte pathogens) but also a post-treatment stable sterile microenvironment. P-3058 is a Terbinafine reformulation that contains the novel excipient hydroxypropyl chitosan as a film-forming agent. In a phase IIB dose-finding, multicentre, randomised, double blinded, vehicle-controlled study (EudraCT No. 2008-002707-10), the subjects were treated with P-3058 regimens of various strength (5% once daily, 10% once daily, or once weekly) vs. the vehicle (once daily or once weekly for 52 weeks). The highest cure rates were achieved by P-3058 10% once daily (68.82% at week 64 and 72.13% at week 76) [23]. Apart from MOB015B and P-3058, BB2603 is another topical Terbinafine-based agent under investigation (NCT04188574). This nano-formula is using polyhexamethylene biguanide (PHMB) as an excipient, and it is administered as a low-velocity spray. Formulations of various strength have been used, aiming to evaluate the potency on the nails and skin (NCT04188574, EudraCT No.2019-002098-68). According to the reported outcomes of a trial studying BB2603-1 (0.01% Terbinafine vs. the vehicle in a phase I/II single-centre, randomised, partially blinded trial; EudraCT No. 2016-001242-25) [40], there was no detectable systemic exposure after a 52-week treatment, the safety and tolerability profile was excellent, and evidence of a mycological cure against dermatophytes was exhibited. In a currently active, double-blinded, phase II study (NCT04188574, EudraCT No. 2019-002098-68), subjects with distal subungual onychomycosis (involving 25–60% of the target great toenail) are randomised and participating in one of the following four arms: BB2603-1 (0.01% Terbinafine), BB2603-3 (0.03% Terbinafine), BB2603-10 (0.1% Terbinafine), and the vehicle (0.3% polyhexanide/20% ethanol/water formulation). The efficacy will be assessed with an early response at week 16 and complete cure vs. the vehicle at week 52. The safety and pharmacokinetics will be also evaluated in the timeframe of 16–52 weeks. A terbinafine-based formulation using poly(pseudo)rotaxanes (PPR) technology to enhance the drug delivery is currently under development [41]. Terbinafine-based PPRs are water-soluble preparations consisting of micelles, α-cyclodextrin, and Terbinafine. The small amphiphilic surfactants solubilise Terbinafine and form 2% (*w*/*v*) gels. It has been recently published that interactions with porcine hooves increase pores and enable Terbinafine to penetrate the structures. PPRs exhibit viscoelastic properties that favour high concentrations in deep layers and prolonged the drug presence at the application site. A subungual insert (HTS-519) has been evaluated in a completed open-label phase II trial (NCT02798380). The subungual placement of a terbinafine-loaded device on days 1, 29, and 57 and estimation of a complete cure rate at week 48 were performed in adults with a dermatophyte infection of one or both great toenails. An open-label phase II study (NCT05135910) to evaluate a Terbinafine subungual gel is currently recruiting subjects with distal–lateral subungual onychomycosis. The gel will be administered once monthly or bi-monthly under the nail plate for a 44-week period of time, and a complete cure of the target toenail will be assessed at week 52.

### 4.4. Topical Amphotericin B

**Figure 7:**
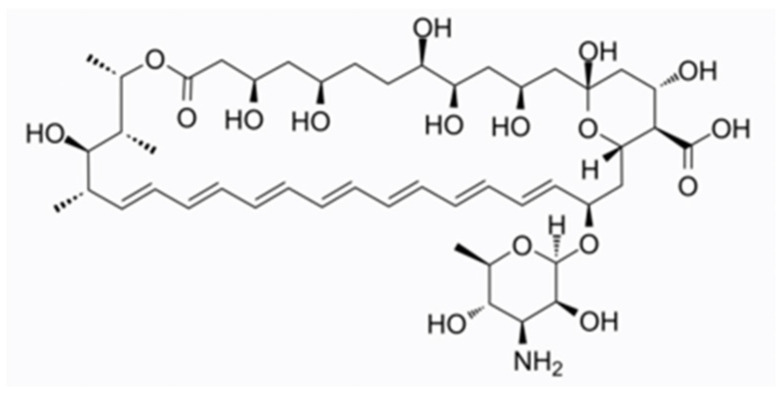
Amphotericin B chemical structure.

Amphotericin B (molecular formula C47H73NO17, chemical structure as seen in Figure 7) is a polyene antifungal agent with a broad fungicidal spectrum produced by Streptomyces nodosus. It binds to ergosterol, causes depolarisation, and alters the permeability of the fungal cell membrane. The subsequent leakage of the intracellular content leads to rupture and, eventually, cell death. A topical Amphotericin formulation (ABL01) for onychomycosis treatment has been recently studied vs. a placebo in a double-blinded, randomised trial (NCT03141840). Adults with distal–lateral subungual onychomycosis of the great toenail (involving less than 50% of the nail) participated in two arms with applications once weekly of either the experimental medical device containing ABL01 or the vehicle for 6 months. There are no published results available. A randomised, double-blinded, phase IV study [24] demonstrated that Amphotericin B was effective when adults with non-dermatophyte onychomycosis were treated with a formulation containing 0.3 mg/mL of the drug in 30% dimethyl sulfoxide cream (DMSO, molecular formula C2H6OS). Dimethyl sulfoxide is a highly polar, water-soluble organic liquid that shows a variety of pharmacological activities [42] and is widely used as a chemical solvent and free radical scavenger. In this trial, the 30% DMSO cream was mixed with the highly lipophilic Amphotericin B in order to enhance the penetration properties of the latter. The treatment was performed before bedtime by applying a pea-sized amount of cream under the occlusion. The vehicle cream served as the control treatment. The clinical status, mycological tests, and safety were assessed during the 36-week intervention and the 36-week post-treatment follow-up periods. A clinical cure was achieved in 70% and 22.2% of the drug and vehicle groups, respectively. The mycological cure rate was 80% in the treated patients and 44.4% in the control arm. All four cases of Fusarium spp. onychomycosis responded to the treatment. However, an extrapolation of the results should not be attempted due to the small number of participants in the study (*n* = 19). DMSO demonstrated some antifungal activity, as it has shown in the past [43]. Furthermore, an Amphotericin B nail lacquer with favourable physicochemical characteristics was produced by using DMSO as the excipient and tested against *Candida* spp. [44]. The lacquer released approximately 90% of the loaded drug content in 3 h, and around 48% of Amphotericin B permeated the ex vivo nail matrix model after 24 h. Moreover, Amphotericin B nanoliposomes have been studied in vitro against 29 archived clinical strains of *T. rubrum* (*n* = 13) and *T. interdigitale* (*n* = 16) [45]. This liposomal Amphotericin B formulation has displayed a lower MIC compared to Amphotericin or the liposomes alone and no evidence of resistance. Nanotechnology may provide an increase in potency without affecting the activity of the drug. In the case of Amphotericin B, nanoliposomes could be an effective way to deliver the drug transungually by using the appropriate particle size and encapsulation.

## 5. Concluding Remarks

Onychomycosis is often an intractable disease with a greatly negative impact on patients’ health, psychosocial behaviour, and quality of life. The prevalence of risk factors for onychomycosis, such as diabetes mellitus, peripheral vascular disease, and immunosuppression, is rising. Secondary complications, aggravation of the underlying pathology (e.g., in psoriasis) [46], and subsequent further exacerbations of onychomycosis create a vicious cycle that affects mainly elderly people [47,48,49]. The demographic shift to a more ageing population in developed countries entails an inevitable increase in comorbidities and polydrug use. High mycological cure rates, pharmacokinetics favouring high concentrations in the nail plate and the subungual space, the mechanism of action mitigating the risk of resistance, minimal adverse events, and recurrence should be among the qualities required from an ideal antifungal for onychomycosis. The therapeutic strategy is usually customised according to the pathogen and the severity of the infection. Nevertheless, safety and resistance will probably become the thorniest issues in the future. Topical treatments are generally considered safe and minimally toxic. Treatment guidelines [50] suggest topical treatments are monotherapies for superficial white onychomycosis (except in transverse or striate infections) and early distal–lateral subungual onychomycosis (except in the case of dermatophytomas), when less than 80% of the nail plate is affected with a lack of lunula involvement, or when systemic antifungal drugs are contraindicated. New and next-generation topical antifungals exhibit high efficacy and show a potential for prolonged biostability in the tissue, even in more severe clinical presentations of onychomycosis. The rationale behind the choice of the appropriate topical treatment should be not only to prescribe an agent with an in vitro documented high potency against a specific pathogen but also to consider the depth and location of the infection on the nail unit. The selection of topical agents for white superficial onychomycosis should take into account the agent’s high affinity for keratin. Similarly, a distal–lateral subungual onychomycosis with extensive subungual hyperkeratosis could respond better to a drug that can penetrate the nail plate efficiently and achieve the highest concentrations deeply into the subungual space and the nailbed. A good insight into pharmacokinetics would assist dermatologists in optimizing the use of topical antifungals and following a more sophisticated therapeutic approach to onychomycosis.

## Figures and Tables

**Table 1:** MEDLINE search strategy (via PubMed; performed on 19 July 2022).

Search Terms (PubMed)	Results *
Onychomycosis	4101
Efinaconazole	156
Efinaconazole [Title/Abstract] AND onychomycosis [Title/Abstract]	121
Tavaborole	83
Tavaborole [Title/Abstract] AND onychomycosis [Title/Abstract]	67
Luliconazole	102
Luliconazole [Title/Abstract] AND onychomycosis [Title/Abstract]	29
Onychomycosis [Title/Abstract] AND topical [Title/Abstract] AND antifungal [Title/Abstract]	333
Onychomycosis [Title/Abstract] AND Efinaconazole [Title/Abstract] OR Tavaborole [Title/Abstract] OR Luliconazole[Title/Abstract]	267
Onychomycosis [Title/Abstract] AND Efinaconazole [Title/Abstract]	237
OR Tavaborole [Title/Abstract] OR Luliconazole [Title/Abstract]	

* Non-human data were excluded.

**Table 2:** Novel topical antifungal agents for the treatment of onychomycosis: Data from RCT records.

Study (Recruitment Status)	Phase	Study Group	Intervention Study Arms (Application)	Primary Endpoint (Timeframe or Timepoint)
**Efinaconazole**				
EH Tche, et al. (2013) [14] NCT00777868 (Completed)	II	Adults mild to moderate toenail DLSO (*n* = 135)	• IDP-108 10% sol * • IDP-108 10% sol • IDP-108 5% sol • Vehicle (one daily application for 36 weeks)	Change from baseline in area of infected nail (time frame: 9 months)
BE Elewski et al. (2013) [15] NCT01007708 NCT01008033 (Completed)	III (two identical multicenter parallel-group studies)	Adults with DLSO (20–50% clinical incolvement), without dermatophytomas or lunula involvement (*n* = 870, *n* = 785)	• Efinaconazole 10% sol • Vehicle (one daily application for 48 weeks)	Percentage of patients who achieve clinical cure (time frame: 52 weeks)
AK Gupta et al. (2021) [16] (Unknown)	IV (single-site)	Adults with mild to moderate DLSO (20–50% involvement) in a TGT (*n* = 101, interim analysis of 47 patients at mo 24)	• Efinaconazole 10% (24 months) • Vehicle (6 mo) followed by Efinaconazole 10% sol (18 months) (one daily application)	• Mycological cure • Effective cure i.e., mycological cure and ≤10% clinical involvement (at week 24)
**Tavaborole (AΝ2690)**				
ME Toledo-Bahena et al. (2014) [17] NCT00679965 (Completed)	II	Adults with mild to moderate (20–60% involvement) onychomycosis of at least one TGT (*n* = 187)	• AN2690 2.5% sol • AN2690 5% sol • AN2690 7.5% sol • Vehicle (one daily application for 3 months; then 3 times a week for 3 months)	Clinical evidence of complete great toenail clearance or at least fungal-clear great toenail growth (“complete” = 5 mm; “partial” = 2 mm), *plus* a negative culture from the treatment-TGT (time frame: 180 days)
BE Elewski et al. (2015) [18] NCT01270971 NCT01302119 (Completed)	III (two identical multicenter parallel-group trials)	Adults with mild to moderate (20–60%) DLSO of the TGT (*n* = 594 and *n* = 604)	• Tavaborole 5% sol • Vehicle (one daily application for 48 weeks)	Completely clear nail and negative mycology of the TGT (at week 52)
AK Gupta et al. (2018) [19]	PSFU (III)	Study group described in Elewski et al. (2015). Analysis of FU at week 60 (post week 52)		• Complete cure of the TGT at week 52 vs. week 60 (completely clear nail and negative mycology) • ASRs and AEs
**Luliconazole (SKX-16)**				
S Watanabe et al. (2017) [20] (Completed)	III	Adults (20–80 years old) with DLSO (20–50% clinical involvement) of the TGT	• Luliconazole 5% solution • Vehicle (one daily application for 48 weeks)	Complete cure rate i.e., 0% clinical involvement *plus* negative KOH direct microscopy
NCT01431820 ** (Completed)	IIB/III	Adults with mild to moderate DSO (*n* = 334)	• Luliconazole 10% sol; (two dosing regimens) • Vehicle; two control arms (one daily application for 52 weeks)	The proportion of subjects who achieve complete cure of the TGT (time frame: week 52)

DLSO: Distal–Lateral Subungual Onychomycosis; FU: Follow-Up; AEs: Adverse Events; TGT: Target Great Toenail; KOH: Potassium Hydroxide direct microscopy; PSFU: Post-Study Follow-Up; DSO: Distal Subungual Onychomycosis * With semi-occlusion. ** Unpublished RCTs listed on https://www.clinicaltrials.gov (accessed on 19 July 2022).

**Table 3:** Topical antifungals under investigation: Data from RCT records.

Study (Recruitment Status)	Phase	Study Group	Intervention Study Arms (Application)	Primary Endpoint
**ME-1111**				
NCT01841996 * (Completed)	I	Adults with moderate to severe DSO of the TGT (*n* = 24)	• ME-1111 solution • Vehicle (one daily application for 28 days)	AEs, application site, plasma concentration, urinary excretion (time frame: baseline to day 57)
NCT02022215 * (Completed)	II	Adults with mild to moderate DLSO (*n* = 304)	• ME-1111 solution, low strength • ME-1111 solution, high strength • Vehicle (one daily application for 48 weeks)	Complete cure (0% clinical involvement of the TGT and mycological cure) at week 52
**NP213 or Novexatin or NVXT**				
NCT02343627 DK Mercer et al. (2020) [21] (Completed)	II	Adults with mild to moderate onychomycosis ** of at least one toe involving 10–50% of the nail (*n* = 47)	• NVXT solution • Vehicle (one daily application on the infected nail and 0.5 mm of adjacent skin for 60 days)	Negative culture after application for 28 days
NCT02933879 * (Completed)	II	Adults with onychomycosis of the TGT (*n* = 184)	• NVXT solution Group A once daily application for 8 weeks; Group B, two 8-week treatment periods separated by a 32-week rest interval • Vehicle Group C, application as per Group B)	Complete cure (clinical and mycological cure) at day 365
**Terbinafine**				
NCT02859519 AK Gupta et al. [22] (Completed)	III	Subjects (12–75 years old) with DSO affecting 20–60% of the TGT (*n* = 365)	• MOB015B 10% sol (Terbinafine HCl) • Vehicle (one daily application for 48 weeks at bedtime)	Complete cure (0% clinical disease and negative mycology) at week 52
NCT02866032 * (Completed)	III	Adults with DSO affecting 20–60% of the TGT (*n* = 452)	• MOB015B solution • Ciclopirox 80 mg/g (one daily application for 48 weeks)	Complete cure (0% clinical disease and mycological cure)
NCT05279846 * (Recruiting)	III	Subjects (12–75 y old) with DSO affecting 20–60% of the TGT	• MOB015B solution • Placebo (one daily application at bedtime for 8 weeks and then once weekly for 40 weeks)	• Complete cure of the TGT at week 52 • AEs (time frame: 52 weeks)
EudraCT No. 2008-002707-10 [23] (Completed)	IIB	Subjects (12–80 years old) with DSO affecting 25–60% of the distal nail plate ***	• P-3058 10% once daily • P-3058 5% once daily • P-3058 10% once weekly • Vehicle once daily • Vehicle once weekly (treatment for 52 weeks)	Responder Rate at the end of FU at week 76 (nail disease area ≤10% of the total plus negative mycology)
NCT02549001 * (EudraCT No. 2015-000561-31) (Completed)	III	Subjects (≥12 years old) with onychomycosis involving 20-50% of the TGT (*n* = 953)	• P-3058 10% solution • Vehicle of P-3058 • Loceryl^®^ nail lacquer • (Amorolfine 5%)	Complete cure of the TGT (negative mycology and totally clear nail)
NCT04188574 * (Active, not recruiting) [EudraCT No. 2019-002098-68] (Ongoing)	II	Adults with DSO affecting 25–60% of the TGT	• BB2603-10 topical spray • (0.1% Terbinafine) • BB2603-3 topical spray • (0.03% Terbinafine) • BB2603-1 topical spray • (0.01% Terbinafine) • Vehicle • (BID for 12 weeks)	Early response for BB2603-10 vs. vehicle at week 16 (clear nail and negative culture)
**Amphotericin B**				
NCT03141840 * (Completed)	N/A	Adults with DSO of the TGT (≤50%) (*n* = 69)	• ABL01 • Vehicle • (once weekly for 6 months)	Clinical improvement (40% reduction in infected area) at 6 months)
NCT03814343 (Completed) C Leeyaphan et al. (2021) [24]	IV	Adults with non-dermatophyte onychomycosis (*n* = 19)	• Amphotericin B • in DMSO 30% • DMSO 30% (application of a pea-sized amount at bedtime and overnight wrapping for 36 weeks)	• Time to mycological cure • Evaluation by the patients with negative mycology

DSO: Distal Subungual Onychomycosis; TGT: Target Great Toenail; AEs: Adverse Events; FU: Follow-Up; DLSO: Distal–Lateral Subungual Onychomycosis; N/A: Not Applicable; DMSO: Dimethyl Sulfoxide; * Unpublished RCTs listed on https://www.clinicaltrials.gov and https://www.clinicaltrialsregister.eu (accessed on 19 July 2022). ** Lunular proximal regions not affected. *** Absence of yellow spikes and/or dermatophytomas without lunula/matrix involvement.

## Data Availability

Not applicable.
